# Incarceration and COVID-19: Recommendations to Curb COVID-19 Disease Transmission in Prison Facilities and Surrounding Communities

**DOI:** 10.3390/ijerph18189790

**Published:** 2021-09-17

**Authors:** Lauren Jeanne Natoli, Kathy Linh Vu, Adam Carl Sukhija-Cohen, Whitney Engeran-Cordova, Gabriel Maldonado, Scott Galvin, William Arroyo, Cynthia Davis

**Affiliations:** 1 Public Health Division, AIDS Healthcare Foundation, Los Angeles, CA 90046, USA; adam.cohen@ahf.org (A.C.S.-C.); whitney.engeran@ahf.org (W.E.-C.); 2 TruEvolution, Riverside, CA 92501, USA; gabrielm@truevolution.org; 3 City of North Miami City Council, Miami, FL 33161, USA; scott@scottgalv.com; 4 California Rehabilitation Oversight Commission, California Department of Corrections and Rehabilitation, Sacramento, CA 95811, USA; wmarroyo@pacbell.net; 5 Department of Psychiatry and Behavioral Sciences, Keck School of Medicine, University of Southern California, Los Angeles, CA 90089, USA; 6 College of Medicine & College of Science and Health, Charles R. Drew University of Medicine and Science, Los Angeles, CA 90059, USA; cynthiadavis@cdrewu.edu

**Keywords:** COVID-19, incarceration, health disparities, racial disparities, public health, disease transmission

## Abstract

Overcrowding can increase the risk of disease transmission, such as that of SARS-CoV-2 (COVID-19), within United States prisons. The number of COVID-19 cases among prisoners is higher than that among the general public, and this disparity is further increased for prisoners of color. This report uses the example case of the COVID-19 pandemic to observe prison conditions and preventive efforts, address racial disparities for people of color, and guide structural improvements for sustaining inmate health during a pandemic in four select states: California, New York, Illinois, and Florida. To curb the further spread of COVID-19 among prisoners and their communities, safe public health practices must be implemented including providing personal protective equipment (PPE) and testing of staff and inmates, disseminating culturally and language appropriate information regarding the pandemic and preventive precautions, introducing social distancing measures, and ensuring adequate resources to safely reintegrate released prisoners into their communities.

## 1. Introduction

The media often highlights the dangers of SARS-CoV-2 (COVID-19) transmission in long-term care facilities and nursing homes; however, prisons are left out of the conversation despite having the most crowded living situations and limited protection measures compared to other facilities. Due to prison environments being filled beyond capacity and a constant influx of prisoners, prisons are highly susceptible to COVID-19 outbreaks when compared to the general population [1]. With the current COVID-19 pandemic, incarcerated individuals, correctional staff, and surrounding communities are particularly vulnerable to contracting and spreading this novel respiratory disease.

By mid-April 2020, rates of COVID-19 in prisons surpassed that of the United States (US) general population [2]. According to August 2020 data, the case rate among prisoners was 3251 cases per 100,000 population, 5.5 times higher than the general population (587 cases per 100,000), and the crude death rate in US prisons (39 deaths per 100,000 prisoners) was also higher compared to that of the general population (29 deaths per 100,000 people) [2]. By November 2020, COVID-19 cases in prisons continued to outpace the general public; confirmed case rates in prisons were 3.7 times the national rate, with 12 in 100 prisoners infected with COVID-19 as compared to three in 100 for the general public [3].

Overcrowding in prisons is the greatest factor contributing to the higher rates of COVID-19 transmission in prisons compared to the general public [4]. In 2018, about 20% of prisons in the US were operating at or above their maximum capacity [3]. Subsequently, there have been 322,769 confirmed cases of COVID-19 among US prisoners with 1846 deaths as of January 2021. For prison staff, there have been 70,658 cases with 113 deaths [3].

To combat COVID-19 transmission during a pandemic, a comprehensive and unified public health effort is desperately needed. This paper looks at the state of COVID-19 in prisons in four US states to report on the disparities and disadvantages experienced by inmates and makes recommendations to combat future COVID-19 outbreaks in prisons and surrounding communities where prisoners are released.

## 2. Materials and Methods

The authors reviewed multiple sources to accurately report on prison conditions, COVID-19 statistics, and recommended guidelines for disease transmission control. Sources included COVID-19 updates from correctional department websites, newspaper articles, first-hand accounts from prisoners, and academic sources. The correctional department websites posted a regularly updated count of tests, infections, recoveries, and deaths for inmates and staff, providing a much-needed level of transparency.

The authors also reviewed public health literature from the Centers for Disease Control and Prevention, the World Health Organization, the US Federal government, universities, and nongovernmental organizations for recommendations regarding COVID-19 mitigation in prisons. Additionally, press releases from local correctional authorities, news articles, and published manuscripts gave a timeline of which public health efforts were implemented and for how long, and the ever-changing understanding of COVID-19 as of December 2020 meant that methods were often altered as new information was learned. The information was organized to create a picture of the COVID-19 pandemic in America’s prison system and provide recommendations on how to curb the crisis behind bars.

## 3. Results

### 3.1. COVID-19 Conditions within Prisons

It is important that the correctional systems have proper testing and treatment in place for people being released to prevent community spread of COVID-19. As reported in the Bureau of Justice’s Annual Survey of Jails, the weekly prisoner turnover rate was 54% in 2017 [5]. Of the 10,570,300 annual admissions in 2017, the estimated average time in jail was only 26 days. This fast turnover rate means significant movement of inmates without social distancing followed by release with missed opportunities to identify and treat COVID-19. Considering this high turnover rate along with the high numbers of prison transfers, prisoners may be exposed to COVID-19 and may not even know they are infected.

One prominent example is San Quentin State Prison. Among California’s 35 prisons, San Quentin had the largest outbreak and was reported as the third largest COVID-19 cluster in the US [6]. According to the state’s Office of the Inspector General, the transfer of infected prisoners from Chino caused the outbreak because these inmates were not tested prior to transfer [6]. In total, California has reported a high number of prison-related COVID-19 cases, leading to 139 COVID-19-related deaths among prisoners [7]. As of 4 January 2021, Los Angeles County alone has reported a total of 4025 symptomatic and asymptomatic COVID-19 cases among prisoners [8].

New York reported that 3109 prison staff, 3101 prisoners, and 170 parolees had confirmed cases of COVID-19, as of 5 January 2021 [9]. Six staff members, 24 inmates, and four parolees have died from COVID-19-related deaths in New York.

Despite precautions, there have been reports from prisoners claiming that they need to be “damn near dying” for the prison staff to provide any COVID-19-related assistance or medical care [10]. Prisoners interviewed about their conditions estimated that about two-thirds of their population have experienced symptoms of COVID-19, but that the staff is slow to provide testing. Furthermore, prisoners say that, while certain areas like the cafeteria are marked for social distancing, many places such as yards, workout areas, showers, and queues are impossible to social distance.

In Florida prisons, 17,537 prisoners and 4389 prison staffers have tested positive, and 191 prisoners have died from COVID-19, as of 5 January 2021 [11]. Florida’s Baker Correctional Facility reported 266 COVID-19 cases among inmates in early August, 42% of the 630 total cases of the entire county at that time [12]. While waiting for test results after the first wave of COVID-19 testing, officers scrambled to place over 1000 prisoners in quarantine, in addition to the 483 inmates already quarantined and 24 inmates already in medical quarantine [12].

The Illinois Department of Corrections reported 8652 total cases of COVID-19 among prisoners with 50 deaths, as of 5 January 2021 [13,14]. In comparison to the most common COVID-19 risks in Illinois—such as poverty, race, public transportation use, and population density—the most significant predictor of COVID-19 infection in Chicago is cycling through the prison system, which is associated with 16% of all documented cases of COVID-19 in the state of Illinois and 16% in the city of Chicago, as of 19 April 2020 [15]. Statistics regarding COVID-19 in all four states are depicted below in Table 1.

### 3.2. Precautions Taken within Prisons

In response to the San Quentin State Prison COVID-19 outbreak in California, the prison installed a large, air-conditioned tent structure able to isolate and treat up to 164 prisoners [6]. This site has since been deactivated after a significant reduction in positive COVID-19 cases [6].

As cases in New York waned from their high in the late spring of 2020, new prison programs were reintroduced with the provision that staff and attendees wear masks at all times, including while in attendance during mental health programs and counseling programs, and while at the library [11]. Visitation resumed in August 2020; however, visiting rooms were configured to reduce capacity and facilitate adequate social distancing. Physical contact between prisoners and visitors is strictly prohibited.

While other states cut back on the number of prisoners, Florida instead opted to place over 14,000 prisoners into isolation for suspected COVID-19 infection [11]. Prison conditions in Florida lack basic precautionary measures against COVID-19, including ventilation, testing, and social distancing. It has been reported that prisoners are denied COVID-19 testing unless they specifically exhibit symptoms of a fever, which excludes the myriad other symptoms related to COVID-19 [10]. As for staff, reports from Florida prisons state that staff who have been exposed to COVID-19 have still been reporting to work despite a May 2020 memo which bars staff with pending COVID-19 test results from working [16]. The combination of poor ventilation, extreme heat, withholding prisoner releases, and low COVID-19 testing sets Florida apart from the other three states discussed in this report.

Despite alarming numbers in Illinois correctional facilities, there is a lack of sanitation and personal protection equipment (PPE; e.g., facemasks and gloves) given to Illinois prisoners. Two prisoners at Statesville Prison in Crest Hill described in letters how prison conditions were facilitating the spread of COVID-19 [17]. Social distancing is impossible with so many prisoners sharing a cell. Prisoners reported that there were no products available to wipe down shared spaces, such as communal phones, in between use [17]. Other reports say that prisoners are cleaning their cells with watered down bleach and dirty rags, while prison staff enter prisoners’ cells throughout the week to do regular shakedowns where the staff touch and potentially contaminate surfaces [17]. While prisoners have limited access to PPE and hand sanitizer, guards and officers are often fully equipped with these products, leaving prisoners feeling vulnerable and unprotected.

### 3.3. Early Release of Prisoners and Re-Entry Support

As of January 2021, California reduced the prison population by at least 10,000, due to the COVID-19 pandemic [18]. In Los Angeles County, prisoners meeting the following conditions are qualified for early release: low risk of reoffending, less than 180 days from the end of their sentence, not serving time for domestic violence or a violent crime, and not required to register as sex offenders [19,20]. While the Illinois Department of Corrections quickly released inmates in the spring of 2020, fewer prisoners are being released in late 2020 than they were pre-COVID-19 [21]. In addition, there are severe age and racial disparities among early release prisoners [21].

Resources dedicated to reintegrating prisoners into society are dwindling with the surplus of prisoners being released early [22]. Although some state and county officials are calling for prisons to release prisoners, there is no extra support provided to ensure safe reintegration into society. As a result, the reentry system is overburdened with sudden releases and is scrambling to find services such as food, transportation, medical care, and housing for released prisoners, many of whom have been exposed to COVID-19 [22,23]. Most jurisdictions partner with community-based organizations to help reintegrate released prisoners; however, these organizations are already overburdened due to the pandemic. One nonprofit organization estimates that it costs 650 USD to transport a single person to a housing facility, including mileage, a meal, PPE, clothing, and employee wages [22]. While it is important to direct more resources to ensure the safe reintegration of released prisoners through testing and healthcare access, local jurisdictions can also help curb the spread of COVID-19 in the local community by halting or drastically reducing admissions [23].

Implementing a unified, nationwide policy is necessary to prevent infected inmates and staff from slipping through the system undetected. Instead, in the cases of many COVID-19 policies, local authorities are left to create their own systems in an unprecedented time, leaving gaps for neglect.

### 3.4. Racial Disparities Regarding Incarceration and Early Release

Discrimination within the prison system leads to health disparities for prisoners of color. In the context of incarceration, people of color are also more likely to be imprisoned compared to White individuals [21]. For example, in 2018, the US Department of Justice’s reported that, within Californian prisons, Black individuals were incarcerated at a rate of 592 per 100,000 population, whereas White individuals were incarcerated at a rate of 187 per 100,000 population [23].

In March 2020, US Attorney General William Barr ordered the federal prison system to release some prisoners to relieve overcrowding in penitentiaries [19]. The planning system for determining who is safest to be released from prison found white-collar offenders generally safer to release. Only 7% of Black male prisoners and 16% of Latino male prisoners were eligible for release compared to 30% of White male prisoners. Furthermore, in Illinois, White prisoners account for only 32% of the prison population, yet they account for nearly half of all pandemic-related releases [21]. In contrast, Black individuals comprise 54% of Illinois prisoners, yet account for only 45% of early releases, and Latinx individuals represent 13% of the prisoner population, yet only account for 10% of the early releases. Furthermore, lawyers and families of prisoners have claimed that the selection process regarding who will be released has not been clear; prisoners who fulfill the guidelines for release are being denied or ignored [21].

## 4. Recommendations

This section summarizes public health recommendations regarding COVID-19 in prisons, which can improve the state of the pandemic in prisons as the virus continues. Prisons and public health officials can pick suitable courses of actions among the following recommendations to ensure that prison facilities initiate more effective primary prevention and mitigation measures to curb the spread of COVID-19 within prison facilities and ultimately in surrounding communities where prisoners are being released.

### 4.1. Enforce Health Precautions

▪ Screen prisoners, staff, and visitors entering and exiting prison facilities for temperature, symptoms, and exposure.
▪ Enforce mask wearing and social distancing.
▪ Encourage staff to stay home when they are feeling ill.
▪ Clean all high-touch areas with increased frequency.
▪ Reduce movement of inmates within institutions and between institutions to the greatest extent possible.

### 4.2. Create Hygienic Environments

▪ Post clear and simple signage throughout prison facilities in English and Spanish that describe proper hand hygiene instructions, PPE use, and social distancing guidelines.
▪ Educate prisoners on COVID-19 prevention in a way that is culturally and linguistically appropriate.
▪ Create proper ventilation and utilize air conditioning at correctional facilities.
▪ Provide prisoners and staff with no-cost soap, hand sanitizer, masks, and gloves.
▪ Increase hand sanitizing stations in prisons, as well as access to running water for handwashing.
▪ Install no-touch hand dryers and trash bins.

### 4.3. Reducing the Prison Population

▪ Release prisoners booked for nonviolent offenses who have served at least 75% of their time, elderly and medically vulnerable prisoners who are not a threat to the community, and prisoners booked on technicalities and minor violations.
▪ Reduce overall prison population size in order to minimize overcrowding and allow for social distancing.
▪ Reduce incarceration and unnecessary face-to-face contact for people on parole and probation.

### 4.4. Ensure Data Transparency

▪ Keep prisoners and staff regularly informed about the number of COVID-19 cases within the facility.
▪ All COVID-19 mitigation signage should be made of clear, simple wording in English and Spanish, with other languages available upon request.

### 4.5. Limitations

This report only focused on prisons in four states, California, Florida, Illinois, and New York, all providing a spectrum of responses and public health measures; however, this may provide a narrow view of the COVID-19 pandemic’s effects considering the spread of the virus in all states. For example, Arkansas, Delaware, Ohio, Oklahoma, and Oregon have reported COVID-19 deaths behind bars that are seven times the rate of the general public, and South Dakota, Arkansas, and Kansas have reported that more than 40% of their prisoner populations have been infected [24]. Furthermore, this paper collected data during the ongoing pandemic from 5 May 2020 to 5 January 2021; COVID-19 case reports and methods of data collection may already be outdated.

## 5. Conclusions

The current state of COVID-19 in the US prison system is abysmal and will only worsen if the public health community does not advocate for change. October and November 2020 reported some of the largest spikes across the US in all settings, signaling that the pandemic must continue to be rigorously addressed as it was in the first few months of the pandemic. The prison system lags on reform, as prisoners are often forgotten. Some of the most basic prevention tools are close to impossible to access in prison—PPE, hand sanitizer, and handwashing—and, with overcrowding, prisoners cannot remain six feet apart from other prisoners and staff. When prisoners are released back into communities, they are without services, programs, and shelter to self-isolate. Public health advocates must depend on local government officials to address the needs of prisoners if the US is going to be able to effectively curb the spread of COVID-19 in US prisons.

Although this report specifically looks at prison conditions under the COVID-19 pandemic, the recommendations and guidelines given can be extended to general disease control protocols. The COVID-19 pandemic has proven that the US prison system is unprepared at properly controlling the spread of a contagious virus; thus, there is a need to establish federal and state guidelines to ensure the future safety of prison inmates, staff, and the surrounding communities where prisoners are released. Furthermore, these federal and state guidelines would further benefit if researchers performed cost–benefit analyses on the precautions taken to curb disease transmission in prisons.

## Figures and Tables

**Table 1:** COVID-19 in prisons as of 5 January 2021—select states.

	California [7]	Florida [11]	Illinois [13]	New York [9]
Number of Prisoners	95,000 *	96,000 *	32,000 *	90,000 *
Prisoner COVID-19 Cases	41,519	15,525	8652	3101
Prisoner COVID-19 Deaths	139	176	50	24
Staff COVID-19 Cases	12,781	2496	3509	3109
Staff COVID-19 Deaths	11	4	1	6

* Approximate. Note: The number of prisoners, prisoner COVID-19 cases, and prisoner COVID-19 deaths were all taken from the respective states’ correctional facilities websites as of 5 January 2021. The staff cases and deaths were taken from the respective states’ correctional facilities websites, in addition to press releases and news articles.
